# Role of Bilingualism and Biculturalism as Assets in Positive Psychology: Conceptual Dynamic GEAR Model

**DOI:** 10.3389/fpsyg.2019.02122

**Published:** 2019-09-26

**Authors:** Xinjie Chen, Amado M. Padilla

**Affiliations:** Graduate School of Education, Stanford University, Stanford, CA, United States

**Keywords:** bilingualism, biculturalism, linguistic awareness, cognitive exploration, assets, positive psychology

## Abstract

Are bilingualism and/or biculturalism good for a person’s positive well being? A growing number of studies have shown different positive outcomes of being exposed to two cultures or speaking two languages respectively, but the benefits of being both bilingual and bicultural have rarely been investigated theoretically or empirically. The purpose of this paper is to summarize the main beneficial outcomes of bilingualism and biculturalism, and to integrate these benefits into a new conceptual framework: *Positive Bilingualism and Biculturalism GEAR model*. The GEAR model suggests that the beneficial outcomes of bilingualism and biculturalism can be systematically classified into four positive dimensions (psychological **G**rowth; cognitive **E**xploration; linguistic **A**wareness; and social **R**einforcement), in which there are dynamic interactions among these four dimensions. The hypothetical GEAR model provides an intricate theoretical approach to understand the potential benefits to an individual of experiencing more than one language and one culture in their life. The proposed model in this research offers a systematic framework for conducting future research to examine whether bilingualism and biculturalism accrue benefits to the individual.

Frequently asked questions among scholars interested in bilingualism are, “How many people are bilingual worldwide? Why is bilingualism important both at the individual and societal level? What do we know about bilinguals that distinguish them from monolinguals? How are bilingualism and biculturalism interrelated? And, under what conditions are both bilingualism and biculturalism equally promoted? It is known that approximately half of the world’s 7.4 billion people are bilingual (Grosjean, 2012). This should not be too surprising since with 193 countries and approximately 6,900 languages and with migration across borders for thousands of years it only makes sense that many people across the globe would find it necessary to speak more than a single language (Grosjean, 2010). Also of importance is that bilingualism can be found among people of all social classes and age groups. Globally, many countries are officially bi/multilingual (e.g., Canada, Belgium, and Switzerland). Although some countries have only one official national language, they may have a considerable number of bilingual speakers (e.g., France and Germany) (Wei, 2008). In one of the largest language surveys, 56% of Europeans (surveyed in 25 different countries) reported that they could use another language besides their mother tongue to communicate (European Commission, 2006).

An important question that must be asked before continuing is: who is bilingual? There is no standard definition of bilingualism (Grosjean, 1982; Anderson et al., 2018). Some researchers have strictly defined bilinguals as individuals who possess native-like control of two languages (Bloomfield, 1933), and who are equally fluent in both languages (Peal and Lambert, 1962). Other researchers hold a broader definition that consider a bilingual to be a person who is skilled in at least in one of the four facets of linguistic competences (i.e., speaking, listening, reading, and writing), even to a minimal degree in a second language. Thus, degree of bilingualism may vary in different skill dimensions (Macnamara, 1967). Other definitions suggest that bilinguals are individuals who can use two languages alternately, or produce meaningful utterances in two or more languages in order to satisfy some communicative need (see review in Grosjean, 1994). The latter points of view may better reflect the reality of bilingualism in society, because it is unusual to identify situations where linguistic skills in two languages are equally employed across the whole range of human discourse. Accordingly, bilinguals mainly use one or the other of their languages to achieve a specific purpose in different contexts, and unequal fluency in their languages is not uncommon (Grosjean, 2012). For our purpose here, we will refer to people with two (or more) language repertoires as bilinguals, regardless of possessing language skills in a second language with varying degrees of proficiency up to full fluency in both languages.

Types of bilingualism have been classified according to various criterion, such as degree of bilingualism (e.g., limited, partial, and proficient bilingual), balanced level (e.g., balanced and non-balanced), mastery skills of reception or of production (e.g., receptive bilingual and productive bilingual), age of language acquisition (e.g., early or late bilingual), sequence of language acquisition (e.g., simultaneous or sequential bilingual), and cognitive processing mechanism between verbal sign and mental image (e.g., coordinated bilinguals, compound bilinguals, subordinated bilinguals) (e.g., Macnamara, 1967; Cummins, 1979). Increasingly research has shown that different types of bilingual experience can affect a bilingual’s developmental trajectory. For example, suggested that those bilingual speakers who learn a second language in a bicultural context might possess a more developed sense of social justice, because of their increased unequivocal empathy for cultural diversity. Lee and Kim (2011) revealed that participants with a higher degree of bilingualism tend to perform better on creative thinking tasks. Researchers have also suggested that learning contexts can directly shape the cognitive abilities of bilinguals (Anderson et al., 2018).

Bilingualism is often important for satisfying a person’s economic, social, educational, and political needs. Which is especially true when there is high interdependence between countries with mutual borders or when speakers of different languages reside within a common geographic area. In the United States, the lingua franca of the dominant group is English, but speakers of other languages who reside in the US must become bilingual, in order to participate fully in social activities and to gain access to educational, medical, and political resources. Thus, for linguistic minorities learning English, while also maintaining the heritage language and culture, is not only a basic survival need but also the vehicle of social status advancement and economic mobility (Citrin et al., 1990). In addition to the immigration-based need above, in today’s global world with its linguistic and cultural diversity, and intercultural contact, many people become bilingual for their work in business, tourism, research, diplomacy, and media in response to the globalization-based demand (García, 2011).

The phenomenon of bilingualism and biculturalism has become increasingly more prevalent and has become the focal interest of research in social and cross-cultural psychology (Benet-Martínez et al., 2006). For some time, researchers have investigated the effects of bilingualism and have identified many linguistic and cognitive beneficial outcomes for bilingual speakers (Bialystok, 1988; Bialystok et al., 2009; Anderson et al., 2018), such as improved metalinguistic skills, better memory and visual spatial skills. On the other hand, different behavioral outcomes have been associated with biculturalism. For example, individuals who identify themselves as bicultural, meaning that they believe themselves to be behaviorally competent in two cultures also possess a set of interdisciplinary outcomes, such as higher levels of creativity, and attentional control in addition to their intercultural competence (Peal and Lambert, 1962; LaFromboise et al., 1993; Padilla, 2006). However, the joint impact for a person showing bicultural and bilingual competence has rarely been investigated systematically (Chen et al., 2008).

## What are the Beneficial Outcomes of Bilingualism?

Early research on bilingual language development in children argued that exposure to two languages could be harmful to a child’s language proficiency and verbal intelligence (Pintner, 1932; Jones and Stewart, 1951). However, Peal and Lambert (1962) showed that earlier research was flawed and that when controlling for confounding factors (i.e., SES, gender and urban-rural contexts), bilingual children performed better on verbal and nonverbal intelligence tasks than their monolingual peers. Since the publication of the Peal and Lambert (1962) study, a large number of empirical studies have demonstrated that bilingual exposure in childhood can have significant cognitive and social advantages. For example, many studies have reported that bilingualism was correlated with better conflict resolution and executive control/selective attention (see review in Bialystok and Viswanathan, 2009). Bialystok has conducted a series of studies on testing the executive functions of bilingual children, and she has reported that children who were raised speaking two languages showed advantages in nonverbal executive control over monolingual children (Bialystok and Martin, 2004; Bialystok and Viswanathan, 2009). Along these lines in a meta-analysis Adesope et al. (2010) found evidence to support the cognitive beneficial effects of being bilingual, including higher levels of attentional control, working memory, and abstract representation skills. Despite a wave of empirical studies that continue to support the notion of a cognitive benefit due to bilingualism across ages (Chung-Fat-Yim et al., 2018; Thomas-Sunesson et al., 2018), some studies have questioned whether there is a bilingual advantage in executive processing (Paap and Greenberg, 2013; DeBruin et al., 2015). For example, Paap and Greenberg (2013) conducted three studies to compare the executive processing ability between bilinguals and monolinguals; their results showed no significant difference between these groups. Hilchey and Klein (2011) based on the results of numerous empirical studies found cognitive advantages due to bilingualism to be more apparent in middle-aged and elderly adults, but very small or even absent effects of bilingualism in children and young adults.

Among these mixed results, researchers further recognized that there is a range of hidden factors that can explain the inconsistent results regarding a bilingual cognitive advantage (e.g., different cultural and linguistic contexts, employing bilinguals with different language learning histories, use of different measurement tools and measuring indicators) (Hilchey and Klein, 2011; Paap and Greenberg, 2013; Torres and Sanz, 2015). For example, Hilchey and Klein (2011) offer an explanation that suggests that a wide range of cognitive advantages (e.g., executive processing) due to bilingualism are observable by using cognitive assessment tools, but not through the traditional nonlinguistic inhibitory techniques used in much of this research. In another example of a complex finding having to do with creativity, Kharkhurin (2010) found that bilinguals demonstrated higher levels of verbal creativity, but lower levels on nonverbal creativity.

Besides studies that have focused exclusively on bilingual cognitive advantages, other advantages of bilingualism should not be ignored. Previous research has also found that early bilingualism has a positive impact on metalinguistic awareness (Cummins, 1978; Bialystok, 1987, 2001b; Ricciardelli, 1992b), psychological adjustment (Chen et al., 2008), and subjective well-being. Furthermore, some research has pointed out that the effects of bilingualism were related to different factors. For example, children with stronger bilingual ability reported better psychological status; metalinguistic awareness performance could vary by the bilingual’s level of proficiency in the language of testing; cognitive task performance could be different based on students’ length of time in a bilingual immersion program (Bialystok and Barac, 2012). According to Cummins (1979) threshold theory, whether bilinguals actually demonstrate positive cognitive effects depends on their competences in both languages. In other words, a bilingual child may only experience the positive cognitive effects if s/he has reached a high level of linguistic proficiency in the languages. Lacking in the child bilingual research literature are studies that seek to understand the cross-dimensional relationships and beneficial outcomes that presumably exist when a child attains proficiency in two languages.

## What are Beneficial Outcomes of Biculturalism?

Similar to bilingualism, early social science views on biculturalism were also negative. For example, in some early research by the noted sociologist Park (1928), he argued that being mixed race and/or bicultural would lead people to suffer from psychological conflict, identity confusion, and normlessness. Concurring with this negative view, Stonequist, who published *Marginal Man* in 1937, maintained the idea that the bicultural person is best captured in the following quote:

*The marginal person is poised in the psychological uncertainty between two (or more) social worlds; reflecting in his soul the discords and harmonies, repulsions and attractions of these worlds…within which membership is implicitly if not explicitly based upon birth or ancestry…and where exclusion removes the individual from a system of group relations.* (Stonequist, 1937, p. 8)

The breakthrough in our understanding of biculturalism did not occur until 1993 with the seminal paper by LaFromboise et al. (1993). In this paper, the authors reviewed the important literature on biculturalism and showed how various models of biculturalism have been used: assimilation model (Gordon, 1978), acculturation model (Padilla, 1980), alternation model (Ogbu and Matute-Bianchi, 1986), multicultural model (Berry, 1986), and fusion model (Weatherford, 2010). Building on these previous models, LaFromboise et al. (1993) recognized that acquiring bicultural competence could be a way to be bicultural without suffering negative psychological outcomes. In order to acquire and maintain competence in two cultures, an individual needs to develop a set of six skills: knowledge of cultural beliefs and values in each culture; positive attitudes toward both cultural groups; bicultural efficacy; communication ability; role repertoire; and a sense of being grounded in both cultures. According to this new perspective, an individual can demonstrate a high level of cultural competence in a second culture while also remaining tied to the culture of origin with the development of these six skills. With the development of greater competency in each culture, the higher the level of biculturalism the person attains and the more enabled they become to effectively manage the challenges of a bicultural existence.

Over time, researchers began to focus increasingly on the positive impacts of biculturalism and found that individuals who possessed behavioral competencies in more than one culture may have a higher capacity to detect and reorganize daily cultural meanings of each group to which they identify (Peal and Lambert, 1962). Psychologically, biculturalism may provide positive coping responses in a racialized society (LaFromboise et al., 1993; Padilla, 2006; Chen et al., 2008). Socially, bicultural individuals who have competence in more than one culture may increase their capability of social competence because by having dual cultural knowledge they are able to demonstrate more social flexibility in response to different social contexts, because of having access to both cultural communities (Feliciano, 2001). It should be noted that not all bicultural individuals experience the full range of positive outcomes; for example, the variations in bicultural identity integration (BII) do have different impacts on their beneficial outcomes, such as creativity (Benet-Martínez and Haritatos, 2005; Saad et al., 2013).

Although accumulated evidence supports the views that bicultural individuals tend to have more advantages socially and culturally, research is limited in showing the benefits in other social/behavioral domains. For example, people who switch more often between different cultural and social frames will have more complex cultural representations, higher ability to detect daily cultural meanings, and possess greater attentional control (Benet-Martínez et al., 2006; Saad et al., 2013). Individuals who identify with both home and host cultures have been found to demonstrate more creativity and to enjoy greater professional success than their monocultural counterparts (Tadmor et al., 2012).

Moreover, some studies have shown that these bicultural positive consequences can interact with each other. Feliciano (2001) found that drawing social resources from both cultures could then benefit bicultural youth for their academic success. However, these interactions have seldom been discussed in the literature. In order to fully capture the dynamics of bicultural experiences, researchers need to examine biculturalism from a multilevel and multidimensional perspective (Chao and Hong, 2007).

## Summary

Previous studies of bilingual and bicultural benefits have, however, been incomplete in three important ways: (1) limited exploration of bilingualism and biculturalism simultaneously for their joint effect on positive assets; (2) an ambiguous picture of the relationships among the positive outcomes of bilingualism and biculturalism, resulting from a large bias by focusing on a few aspects such as the cognitive benefits of bilingualism and positive social outcomes of biculturalism while ignoring other possible benefits; and (3) a dearth of studies designed to investigate how bilingualism and biculturalism are linked with each other.

## The Need for a New Positive Theoretical Framework

To address the research gap described above it is of great importance to (1) combine the assets attributable to bilingualism and biculturalism in order to gain an overarching perspective of how they were work in unison; (2) examine the benefits of bilingualism and biculturalism in a more complex way, by extending how these benefits possibly influence other behaviors, which lend themselves to classifying these additional assets in a systematic way; and (3) explore the links and interactions among all positive assets. To accomplish these objectives, we propose to use tenets from positive psychology to offer a framework for uniting bilingualism and biculturalism.

Positive psychology (PosPsy) aims to investigate realistic ways of fostering more well-being in individuals and communities by promoting positive traits, happiness, and flourishing (Seligman and Csikszentmihalyi, 2000). Through PosPsy, researchers have sought to explore and promote the strengths and positive assets of human existence, instead of solely using psychology to prevent or intervene on social problems and weaknesses. Several asset-based models have been proposed. For example, the broaden-and-build theory by Fredrickson (2001) suggested that four resources (cognitive, psychological, physical, and social) can be developed by experiencing positive emotions that can enable the individual to become a better self. Keyes and Haidt (2003) also proposed three assets that lead individuals to flourish: emotional, psychological, and social well-being. Park et al. (2004) have applied the strength-based approach to investigate human well-being by identifying 24-character strengths (e.g., curiosity, social intelligence, gratitude, hope, optimism, humor, mercy, forgiveness, and sense of purpose) that can enhance individuals’ life satisfaction. Consistent with the aim of PosPsy, the new theoretical framework that we propose here seeks to capture the important components of being bilingual and bicultural through new positive psychological lens. This new theoretical framework employs a positive psychological asset-based perspective to explore the beneficial outcomes associated with bilingualism and biculturalism, and to help language and culture researchers better understand the assets of bilingualism and biculturalism in a positive, complex, holistic, and dynamic way.

## The Gear Model

**Figure 1:**
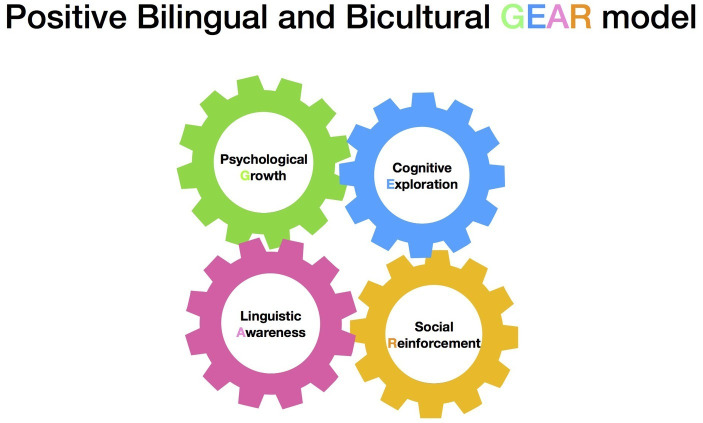
It depicts the GEAR model and the interconnectedness between the four components that comprise bilingualism and biculturalism.

This paper outlines a new, multidimensional model that envisions bilingualism and biculturalism as multilayered and interacting with each of its components. The Positive Bilingual and Bicultural GEAR model consists of four components: psychological, cognitive, linguistic, and social resulting in *psychological **G**rowth*; *cognitive **E**xploration*; *linguistic **A**wareness*; and *social **R**einforcement*. The model is shown below in Figure 1.

## Description of the Complex and Dynamic Gear Model

In this section, we provide a description of the assets associated with each component of the GEAR model.

### Psychological Growth

There is a growing body of literature that indicates that bilingualism is closely related to one’s psychological development. First, language learning is viewed as a key factor associated with self-related constructs [e.g., self-confidence, self-concept, self-esteem, self-efficacy, and identity (Duff, 2007; Mercer, 2011)]. As a result, bilingual individuals may have different self-structures in their different languages; the use of a particular language is associated with the relevant specific cultural frame in which the language is embedded. This cultural belief system may promote different self-concepts depending upon the culture (Wang et al., 2010). Mercer (2011) further suggested that a bilingual learner’s self-concept could be a complex, multilayered, multidimensional network of interrelated self-beliefs; it is dynamic and also relatively stable according to its types and forms.

The language we use as a mother tongue connotes who we are culturally and linguistically and this provides the nurturance and stability necessary for a person’s healthy development and fulfillment (Padilla and Borsato, 2010). Similarly, a person who is bilingual and/or bicultural reflects a person who identifies as a member of two linguistic and/or cultural groups. An individual’s self-concept is closely related to their language group emotionally and socially (Fielding, 2015). Benet-Martínez et al. (2006) show that the experiences that a bicultural person has are often determined by their skin color and phenotype, learned observable behaviors, their accent, and how these are received by members of either of their two cultures. This also has the potential of affecting their self-concept. In line with this, bicultural identity research has found identity to be positively related to self-concept and negatively related to psychological discomfort (de Domanico et al., 1994; Clark and Flores, 2001).

Second, a large body of bilingual and bicultural research has shown that learning a second language could contribute to one’s psychological well-being. For example, Oxford and Cuéllar (2014) found that learning Chinese can help Hispanic students to self-discover, and to enrich positive emotions associated with activity engagement, relationships, meaning, and accomplishment. In other words, learning as a second language can be viewed as a powerful life experience of openness to new a culture and its people, history, values, and artistic expressions. Furthermore, highly strategic language learners may reflect more positive outcomes related to well-being, such as resilience and hope (Oxford, 2014). Historically, the bilingual person has served as the intermediary through translation between monolingual members of different cultures. Accordingly, the bilingual and bicultural person experiences both personal and communal recognition for the ability to navigate across linguistic and cultural barriers in ways that provide positive experiences and psychological growth.

### Cognitive **E**xploration

Accumulating research on bilingualism shows that bilingualism is an experience that has significant consequences for cognitive performance, such as conflict resolution (Bialystok et al., 2009), mental flexibility, and creative and divergent thinking skills (Lazaruk, 2007; Adesope et al., 2010). As explained by Lubart (1999), language, as a vehicle of culture, can shape creativity. Bilinguals may have a greater diversity of associations to the same concept and diverse ways to encode and access knowledge, so that they may have a more flexible and creative approach to know the world. It should be noted that several factors can lead to different degrees of creativity among bilinguals, such as second language proficiency, age of second language acquisition, input (e.g., formal vs. informal) of second language, pedagogical content of the second language, and a bilingual’s personality characteristics. As reviewed by Ni (2012), a higher level of bilingual creativity is related to greater second language proficiency (Ricciardelli, 1992a; Lee and Kim, 2011), earlier age of acquiring the second language (Cushen and Wiley, 2011), more appropriate selection of pedagogical content in language class (Fleith et al., 2002), and an extrovert personality (Leung and Chiu, 2008).

Besides creativity, a recent study also found greater divergent thinking abilities among a group of foreign language learners, when compared to non-foreign language monolingual peers (Ghonsooly and Showqi, 2012). Moreover, these cognitive advantages may further contribute to their academic outcomes. Collectively these studies support the initial findings of Peal and Lambert (1962) that showed French-English bilingual students in Montreal had better academic performance than their French or English monolingual peers.

In terms of biculturalism, bicultural individuals may have higher levels of cognitive complexity than their monocultural counterparts. Knowledge of more than one culture may increase people’s ability to detect, process, and organize everyday cultural meaning. Accordingly, the bicultural individual may be more able to flexibly shift their behaviors in culturally appropriate ways depending on the cultural environmental context (Chao and Hong, 2007). Moreover, different types of biculturalism may be related to different outcomes. For example, bicultural individuals who perceive their two cultural orientations as somewhat conflicting and incompatible think in cognitively more complex ways about their cultures than those who perceive their two cultural orientations as compatible. Benet-Martínez et al. (2006) have suggested that if a bicultural person perceives their two cultural orientations as conflicting and dissociated, they might make more effort to encode the cultural information than those who perceive their cultural identities as more integrated. More complex cultural representations among biculturals with lower levels of cultural integration suggest that the development of cultural schemas is of necessity richer in content, more differentiated and integrated. Further research is required to better understand the dimensions under which bilingual and bicultural individuals must develop their linguistic and cultural competencies.

### Linguistic **A**wareness

Linguistic awareness entails an understanding of how a language works and as such is divided into numerous subcategories such as: phonological, semantic, syntactic, and pragmatic awareness. With development, the learner becomes increasingly able to engage in a cognitive process of thinking about the use of language – known as “metalinguistic awareness.” With metalinguistic ability, the learner is able to see language as a code that is separated from the symbolic meaning of words. Metalinguistic awareness is the conscious understanding of the properties of language and how language is used to communicate with other interlocutors through either speech or written language. As children learn to speak their home language, they become increasingly more capable of monitoring and controlling their use of language. This becomes crucial in the learning of two languages by child bilinguals or in the learning of a second language once a first language is established.

Accumulating evidence has shown that bilingual individuals perform better on metalinguistic awareness tasks than their monolingual peers in terms of word awareness (Cummins, 1978), grammatical (syntactic) awareness (Galambos and Hakuta, 1988; Galambos and Goldin-Meadow, 1990), and phonological awareness (Rubin and Turner, 1989). These linguistic advantages were found not only among fully functional bilingual children but also children who had some bilingual immersion program experience and were still in the process of becoming bilingual (Bialystok et al., 2014). Moreover, these advantages may have differential effects on different linguistic domains. For example, learning two languages may increase one’s “ear” or phonological awareness for regularities of form, but have no effect on grammatical or syntactic awareness for regularities in syntax among early bilingual children (Galambos and Goldin-Meadow, 1990).

Previous research provides evidence that bilinguals’ level of metalinguistic awareness varies by language proficiency among bilingual speakers (Bialystok et al., 2003) and by different combinations of bilingual types, such as Spanish-English or Chinese-English bilinguals (Bialystok et al., 2003). Research on bilingualism has also shown that a bilingual’s metalinguistic awareness can impact their language performance in both languages, such as reading and vocabulary knowledge (Cheung et al., 2010), as well as their ability to acquire a third language (Thomas, 1988). The issue here is that once we learn a second language we have a better grasp of how languages work and as a consequence have a deeper understanding of the metalinguistic skills that differentiate languages. Another feature of bilingualism is that the person who is proficient in two or more languages possesses a richer appreciation of subtleties between how languages are used in different contexts. A common practice among bilinguals that demonstrates the full impact of linguistic awareness is code-switching. According to Luna and Peracchio (2005), code switching is a way of communicating with language selection, and the change of language choice reflects the shift of individual’s social identity. Because bilinguals possess a richer store of linguistic assets or “codes” (e.g., languages, dialects, styles, and registers), bilinguals possess higher levels of sensitivity for selecting codes according to different linguistic and cultural contexts. The ability to switch linguistic codes with ease often observed among bilinguals reflects the speakers’ awareness of the functions of language variation in social interaction (Nilep, 2006). Therefore, linguistic and metalinguistic awareness are assets that many bilinguals demonstrate on a daily basis in their home and in their community of other bilingual speakers. Code-switching has been studied by sociolinguists who have shown that the rich switching between languages in bilingual dialogue is not random, but rather is a manifestation of a better understanding of the functional use of languages. This is counter to the earlier view that language mixing or Code-switching was a marker of a speaker who did not have command of either language and was used as an argument for not encouraging early child bilingualism (Alderson et al., 1997).

Moreover, a person’s metalinguistic ability can interact with their cognitive abilities in varying ways (Bialystok et al., 2014). For example, Bialystok (2001a) has shown that bilingualism can enhance a child’s metalinguistic awareness, especially in tasks where a high level of executive control is required. In other words, a bilingual’s level of executive control may also determine their metalinguistic ability on a linguistic task.

### Social **R**einforcement

Research on the social effects of bilingualism has consistently shown that speaking more than one language increases one’s ability to respect more linguistic and racial diversity (Dagenais et al., 2008; Little, 2012; Parys, 2015). This should not come as a surprise because languages are intertwined with cultures. Proficiency in two or more languages demonstrates that the person has a firmer grasp of diversity on different levels because the ways in which people of different cultures interact are represented in how language is used in social discourse. In addition, the person who is proficient in two or more languages has more opportunities to interact with more diverse social and cultural groups than his/her monolingual counterpart, who is more restricted in social contacts. Language brokering is a prevalent language contact phenomenon in bilingual children who often are called upon to serve as informal translators for their family and community members (Shannon, 1990). This brokering or translating experience serves to reinforce the social connection within the bilingual heritage ethnic group, as well as between the heritage and mainstream ethnic groups. Language-brokering activities create opportunities for parents and other adult family members to teach their heritage culture, practices, values and traditions to their bilingual children. This activity fosters bilinguals’ ethnic identity and their sense of belongings within their family’s ethnic group (Weisskirch et al., 2011). Children who serve as bilingual language brokers play an important role in easing their families’ connections to the mainstream society by opening their families’ access to resources and information in various domains (e.g., medical, educational, and work; Orellana et al., 2003). Moreover, for bilinguals themselves, the task of language brokering increases their level of social self-efficacy and acculturation in mainstream society (Love and Buriel, 2007). The ability to serve as a language broker is an another example of an asset brought on by bilingualism since the ability to serve as a linguistic and cultural broker reinforces the social connection by bringing together people of different ages and backgrounds who otherwise might not interact with each other and thereby facilitating communication in a positive way across language barriers within and between ethnic groups.

As observed above, previous research has suggested the potential interaction between these four components (i.e., psychological growth, cognitive exploration, linguistic awareness, and social reinforcement) in general. For example, self-esteem contributes positively to one’s creative and divergent thinking (e.g., Deng and Zhang, 2011; Cantero et al., 2016; Wang and Wang, 2016). However, individuals with higher levels of explorative cognition, such as cognitive flexibility or divergent thinking skills, tend to experience more self-esteem and life satisfaction (Kim and Omizo, 2005). Similarly, in the context of bilingualism and biculturalism, deep experiences with two languages and cultures enable bilinguals and biculturals to express greater cognitive flexibility, allowing them to better adopt and adapt to challenges of living in multicultural contexts, which in turn promote a more wholesome psychological development. In return, individuals with greater resilience and positive psychological perception toward self, might be more willing to explore cognitively and socially, rather than hold back because of the concern over failure and social rejection (e.g., Zeigler-Hill et al., 2015).

Furthermore, research has demonstrated the existence of a relationship between linguistic awareness and social reinforcement. For example, bilingual individuals with a higher sense of linguistic awareness are more capable of switching the appropriate registers based on different linguistic and cultural environmental contexts and in so doing are better able to engage in interpersonal communication across linguistic and cultural boundaries that reinforce the connections within and between different communities (e.g., Love and Buriel, 2007).

In recent years, language teachers have also begun to couple second language instruction with service learning, where students can use their newly acquired language in an authentic context, which increases a learner’s civic responsibility (O’Brien, 2017) and social link with the global world (Caldwell, 2007). Learning a second language allows students to make more connections with a broader array of people across the globe (Melin, 2013) while also enriching their sociocultural ability (Wang et al., 2010). Importantly, Sung (2016) has shown that second language learners with high integrative motivation for learning a new language display open-minded attitudes toward speakers of the other language. These learners are highly motivated to seek out people, who speak the target language they are learning. In addition, research shows that these learners are also interested in the culture associated with the new language. They want to learn the language for the sake of the language and the culture and not for an instrumental motive like enhancing their employability. These learners may have significant others such as close friends or family members who speak the language, with heritage language learners typically having a particularly strong integrative motivation for learning the home language. Studies have found that language learners who possess integrative motivation are more successful language learners and attain higher levels of proficiency in the new language than those who merely want to learn the language for some instrumental advantage such as gaining college admission or securing employment (Gardner and Lambert, 1972). Furthermore, higher levels of motivation in second language were also related to one’s higher self-perception of global competence (Semaan and Yamazaki, 2015).

Because language is a vehicle to understand culture, speaking a second language and experiencing its culture allow students to better understand and appreciate other cultures. Accordingly, bilinguals are typically more welcoming of diversity, whether cultural or linguistic, than their monolingual counterparts. This awareness of difference and diversity may contribute to decreasing stereotypes and implicit bias between groups of people while allowing for the development of rich interpersonal relationships that extend beyond social or cultural boundaries (Forsman, 2010). Bilingual and bicultural individuals have this capacity. Their openness to diversity allows them to enjoy and experience higher levels of intercultural communication with more people than individuals who must understand their world though a single language filter. As O’Brien (2017) suggests, bilingual speakers tend to demonstrate higher empathy and are more likely to advocate for social justice. In addition, individuals who learn a second language in a bicultural context may also have a more developed sense of social justice because of their increased empathy for culturally diversity.

In short, bilingualism and biculturalism serve as additional positive and meaningful assets for connecting with a broader community of people from different cultures and linguistic backgrounds. As a result being bilingual and/or bicultural can contribute to reinforcing one’s social bond and build positive relationships with people from different cultural backgrounds.

## Uniqueness of Gear Model

There are many benefits associated with bilingualism and biculturalism; however, what is lacking is a coherent framework within which to understand the relative importance of these different types of benefits, and the possible interactions among their assets. Based on previous research, we propose our GEAR model as a useful conceptual tool for researching the unique assets of being bilingual and bicultural within the framework of three unique characteristics as discussed below.

First, the Gear Model evolved as an attempt to resolve the disorganized situation that currently exists in the literature on the benefits of bilingualism and to do so by using a positive asset based model. This model offers a different lens through which to study and to understand the benefits to individuals who are bilingual and/or bicultural. The goal is to classify and systematize these assets into a framework that enables us to better discuss the advantages of bilingualism and biculturalism. Although it is commonly known that language and culture are closely interrelated, we use language to communicate our thoughts and feelings, to connect with others, to identify with our culture, and to understand the world around us. Little research investigates the intricacies between bilingualism and biculturalism (Chen et al., 2008). The hope is that the GEAR model will serve as a bridge between bilingualism and biculturalism research by summarizing their common and differential effects on human behavior.

Second, the core of the model is to understand the beneficial outcomes of bilingualism and biculturalism using a positive psychological framework. The GEAR model combines bilingualism and biculturalism and examines their assets from an overarching positive perspective (i.e., psychological growth, cognitive exploration, linguistic awareness, and social reinforcement) that focuses on aspects of the human condition, which culminates in personal happiness and the feeling of flourishing (Linley et al., 2006). In other words, an understanding of the interdependence between bilingualism and biculturalism will result in a higher level of fulfillment and accomplishment since it is not necessary to make a decision favoring one language or culture over the other.

Third, our GEAR model extends the discussion of possible beneficial outcomes related to bilingualism and biculturalism in a broader way. The psychology surrounding biculturalism and bilingualism is a complex phenomenon that has evolved over time from a past negative perspective to a more recent focus that emphasizes the advantages of bilingualism and biculturalism. In addition, earlier research that focused on single impacts of bilingualism or biculturalism in certain domains, but the GEAR model provides a positive and holistic framework to look for the beneficial outcomes in a more complex way, as well as the links between the four proposed outcomes. The GEAR model aims to consider bilingualism and biculturalism as resourceful agents with their respective associated assets interacting with each other dynamically and in return promote a more positive bilingual and/or bicultural individual. Ideally, this dynamic perspective can result in original insights and a more comprehensive understanding of how bilingualism and/or biculturalism affect the individual in positive and psychological growth enhancing ways.

## Conclusion

In sum, the GEAR model proposed here covers a broad array of literature on the positive benefits of bilingualism in the cognitive and linguistic domains of everyday life. While we acknowledge that not every study supports all the tenets of the GEAR model, we believe that overall, the literature is quite robust in favoring the positivity surrounding bilingualism and biculturalism. Hence, the GEAR model is a viable conceptual framework and tool to investigate and highlight the positive effects of being bilingual and bicultural, which takes relevant benefits into account. Based on the proposed model, we believe that bilingualism and biculturalism can lead to a set of interrelated personal assets in psychological, cognitive, linguistic, and social domains that enrich the lives of people because they have multiple channels of communication and lenses through which to interpret their daily experiences. We maintain that individuals who are bilingual and bicultural and who take an active role in the process of becoming bilingual and bicultural are more likely to experience a richer set of positive life outcomes (e.g., happiness, life satisfaction) than individuals who remain tied to a single language and culture or who give up a heritage language/culture in favor assimilating into a dominant language/culture.

## Future Research

Future research in bilingualism and biculturalism might consider applying the GEAR model as a theoretical framework to systematically analyze the beneficial intersections of being bilingual and bicultural. Empirical studies are needed that examine in methodologically sound ways the precise positive outcomes identified in the four components proposed by this model and which do so across different cultures where bilingualism and biculturalism are the norm or not. Several research paths are possible. In terms of the separate effects of bilingualism and biculturalism, it would be interesting to investigate the extent to which cognitive and psychological outcomes differ between bilingual and bicultural individuals. For example, researchers might explore the same outcomes by comparing bilingual subjects who are not bicultural (e.g., Catalan Spaniards who speak both Catalan and Spanish, but who are culturally Spanish), and those who are bicultural but not bilingual (e.g., many later generation Latinos in the USA). This might provide a clearer picture of the unique contribution of bilingualism and biculturalism in people’s life.

In terms of the joint effects of bilingualism and biculturalism, more research is needed to identify the extent to which different combinations of bilingualism and biculturalism are linked and how the beneficial outcomes may be attributable to each, such as higher bilingualism with lower bicultural identity or vice versa. For instance, in a multicultural country with people of mixed cultural heritage, early/native bilinguals are usually bicultural such independent with simultaneous bicultural bilingual identity could demonstrate beneficial effects in line with the GEAR model, which may be different from bilinguals who merely acquire their second language in a monocultural context. Thus, the proposed GEAR model could provide researchers with a framework to explore the main dimensions of the positive outcomes across different types of bilingual and bicultural group configuration. Future studies that are designed to provide empirical support across linguistic and cultural contexts to triangulate the positive benefits of bilingualism and biculturalism in a more complex and in-depth way are strongly encouraged.

Similar to the idea proposed by Ortega (2013), bilingual and bicultural researchers have been very good at asking what the other sciences can do for the study of language and culture. As the field enters the twenty-first century, it is time now to ask how our deeper understanding of bilingualism and biculturalism can contribute to how language and culture are taught in schools, why people hold strong allegiance to heritage languages and cultures, how better second/foreign language teaching and learning can contribute to enhancing intercultural communicative competence, and how second/foreign language teaching can do more for individual learners beyond the simple linguistic acquisition of a new language through a greater emphasis on the culture behind the language. By focusing on the benefits of bilingualism and biculturalism together, we are in a better position to reflect on how to assess what constitutes good second/foreign language teaching and learning in a more complete and intricate way.

## Author Contributions

XC designed the research and drafted the paper. AP designed the research and revised on the paper.

### Conflict of Interest Statement

The authors declare that the research was conducted in the absence of any commercial or financial relationships that could be construed as a potential conflict of interest.
