# Corrigendum: Role of bilingualism and biculturalism as assets in positive psychology: conceptual dynamic GEAR model

**DOI:** 10.3389/fpsyg.2024.1513187

**Published:** 2024-12-09

**Authors:** Xinjie Chen, Amado M. Padilla

**Affiliations:** Graduate School of Education, Stanford University, Stanford, CA, United States

**Keywords:** bilingualism, biculturalism, linguistic awareness, cognitive exploration, assets, positive psychology

In the published article, the references for Crosbie, V. (2014) and Tran, T. V. (1995) were incorrectly included and should be removed from the reference list.

The authors apologize for this error and state that this does not change the scientific conclusions of the article in any way. The original article has been updated.

